# Predictors of Engagement in Community-based Residential Mental Health Rehabilitation: Modelling of a cross-sectional Statewide Benchmarking Dataset from Queensland, Australia

**DOI:** 10.1007/s10597-025-01512-6

**Published:** 2025-09-10

**Authors:** Olivia Falvey, Donna Jones, Terry Stedman, Stephen Parker

**Affiliations:** 1 Queensland Mental Health Benchmarking Unit, West Moreton Hospital and Health Service, Wacol, Australia; 2 The University of Queensland, Herston, Australia; 3 Queensland Mental Health Benchmarking Unit, West Moreton Hospital and Health Service, Wacol, Australia; 4 Metro North Mental Health, Herston, Australia

**Keywords:** Psychiatric rehabilitation, Residential services, Engagement, Psychotic disorders

## Abstract

Engaging residents with the support available at community-based residential mental health rehabilitation facilities is an ongoing challenge for health services. This study explored factors associated with residential rehabilitation engagement across Queensland, Australia through regression modelling of cross-sectional data from a statewide benchmarking activity completed in 2023 (*n* = 208). The Residential Rehabilitation Engagement Scale (RRES) assessed each resident’s rehabilitation engagement. A broad range of potential predictors were considered, including resident and unit-level variables. Only 45.2% of residents had an average RRES score consistent with being engaged with rehabilitation support usually or always. Higher levels of rehabilitation engagement were significantly associated with lower levels of psychosocial disability (*B* = − 0.413, *p* < .001), length of treatment (*B* = − 0.165, *p* = .008), care under the integrated staffing model (*B* = 0.156, *p* = .012), higher staff recovery knowledge and attitudes (*B* = 0.138, *p* = .037), and physical illness or disability (*B* = 0.129, *p* = .045). In conclusion, engagement in residential rehabilitation was associated with both resident and staff factors. The observation that engagement was higher where unit staff endorsed recovery knowledge and attitudes, and under the integrated staffing model is important. This suggests potential modifiable service characteristics that may support improved rehabilitation engagement in the future.

**Supplementary Information:**

The online version contains supplementary material available at 10.1007/s10597-025-01512-6.

## Introduction

Engaging residents with the support available at community-based residential mental health rehabilitation facilities is an ongoing challenge for health services (Kayes et al., 2015). These rehabilitation services typically support people experiencing severe and persisting mental illness, most commonly schizophrenia, to increase their independence and participation in the community (Parker et al., 2019a, b, 2023). Contemporary services typically provide high staff support, emphasizing helping residents ‘move on’ to more independent accommodation and reduced reliance on clinical care. Reassuringly, evidence is increasingly available indicating that positive outcomes are being achieved for contemporary residents (Parker et al., 2020; Slade et al., 1999; Tolonen et al., 2024; Vanzetto et al., 2021). However, building engagement in longer-term rehabilitation settings remains a challenge (Kayes et al., 2015; Meaden et al., 2012, 2014); many residents do not participate in the programs and interventions offered. A study published in 2014 based on data from the United Kingdom (UK) found that only a third of those residing in community-residential services were actively engaged (Meaden et al., 2014). Similar challenges are faced in the Australian context, with a recent study finding more than a third of people admitted to community-based residential mental health rehabilitation services exit before the completion of planned care (Arnautovska et al., 2021).

The existing research on engagement with mental health rehabilitation services is limited. This literature is mainly focused on describing service-user behaviors perceived as problematic by staff or the characteristics of their non-engagement (Arnautovska et al., 2021; Kayes et al., 2015; Meaden et al., 2012, 2014). Some criticism has been directed at the focus on resident characteristics, behaviour, attitudes, and beliefs when seeking to understand the challenge of rehabilitation engagement. It is also important to consider the impact of staff and service delivery structure as potential determinants of rehabilitation engagement and outcomes, as well as the co-constructed nature of engagement between staff and residents (Bright et al., 2015; Killaspy et al., 2015). For example, a single Australian study based on 2021 data suggested that residents’ agreement and active participation at community-based rehabilitation were higher in units where peer support workers (PSWs) were the majority workforce component (Karan et al., 2022).

The current study explores factors associated with engagement at community-based residential rehabilitation services across Queensland, Australia. A broad range of potential predictors was explored, including staff, service, and resident factors. As an exploratory analysis no hypotheses were pre-specified. The findings of this study are expected to enhance the understanding of the factors associated with rehabilitation engagement to guide future interventions to enhance participation in evidence-based psychosocial rehabilitation programs.

## Methods

This study reflects a secondary analysis through multiple regression modelling of a statewide cross-sectional dataset collected by the Queensland Mental Health Benchmarking Unit (data collection: 06/03/2023 ‘census date’). All Queensland Community Care Units (CCUs) participate in a biannual benchmarking exercise involving cross-sectional data collection of resident and service characteristics. The benchmarking provides a process for comparing services to identify areas for improvement and monitor the impact of quality improvement initiatives (Shepherd et al., 2010).

A formal application for exemption for the requirement of ethics approval was supported by the West Moreton HHS Human Research Ethics Committee (EX/2024/QWMS/105695) under the National Statement on Ethical Conduct in Human Research 2023. The study is reported following consideration of the STROBE reporting guidelines for cross-sectional studies (Von Elm et al., 2007).

### Study Context

The Community Care Unit (CCU) model is Australia’s most common community-based mental health rehabilitation service type. Contemporary CCUs provide mental health rehabilitation of the Transitional Residential Rehabilitation type (Parker et al., 2019a), that are similar to the STAX-SA Type 2 services (McPherson et al., 2018) (i.e., staff on-site, high support, strong emphasis on move-on, congregate setting) available in the United Kingdom and several European countries. CCUs provide assertive rehabilitation support over 6 to 24 months, focused on living skills development and community integration. Most of the people referred to these units will have a diagnosis of schizophrenia or a related psychotic disorder and have complex care needs (Parker et al., 2019a, b, 2020, 2023; QMHBU, 2023).

The public health system in Queensland, Australia, operates fourteen CCUs with a combined capacity of 300 beds. All support delivered at the CCU is provided at no cost to residents, and they pay a heavily subsidized rental fee. All the CCUs have a shared model of service. However, individual CCUs operate different staffing configurations regarding the proportionate availability of nursing, lived experience (PSW), and other non-clinical support staff (Karan et al., 2022). Under the traditional ‘clinical staffing’ approach, nursing roles comprise most of the workforce. Under the ‘integrated staffing’ approach, PSW roles predominate, and under the ‘partnership staffing’ approach, the health service works collaboratively with a non-government organization, and there is an increased availability of non-clinical support roles. Regardless of the staffing configuration, the CCUs model emphasizes multidisciplinary clinical support, including medical, nursing, occupational therapy, psychology, occupational therapy, and social work roles.

Core psychosocial interventions provided under the CCU model include Cognitive Behavior Therapy, Cognitive Remediation, Family education and support, Psychoeducation, and living skills development. Except for ‘living skills development’, these psychosocial interventions are classified in the ‘Royal Australian and New Zealand College of Psychiatrists clinical practice guidelines for the management of schizophrenia and related disorders’ as having ‘Level 1 evidence’ for supporting people living with schizophrenia (Galletly et al., 2016). In the Queensland CCU context, vocational rehabilitation support is generally delivered through external providers. Links to further information about the nature, experience, and variation in the CCU service delivery in Queensland is provided in the Supplementary Materials.

### Participants and Sampling

The 2023 CCU Benchmarking round provided a complete sampling of residents at Queensland CCUs on the census date. Demographic, treatment, and staff-rated engagement data were provided and/or extracted by trained unit staff, and the benchmarking team checked the integrity of the data. Additionally, unit staff provided all residents with the opportunity to complete a self-report survey, and all staff were also invited to complete a staff survey. For this study, residents with a length of stay (LOS) of less than six weeks were excluded from the analysis due to the likelihood that the focus of support would be assessment rather than rehabilitation (*n* = 35/257). A small number of residents with LOS exceeding three years were also excluded (*n* = 9/257, LOS range 3.03–19.90 years); these residents reflect atypical cases as their care has exceeded the maximum duration designated in the model of service by 50%.

### Outcome Focus

The outcome focus was engagement with the residential rehabilitation service for each individual resident using the Residential Rehabilitation Engagement Scale (RRES) (Meaden et al., 2012, 2014). This staff-rated instrument was developed in the UK to assess the engagement of people experiencing psychosis in clinically operated residential mental health rehabilitation care. Responses to the 16-items are provided on a 5-point Likert scale (items scored 1-to-5 with higher scores equating to higher engagement). Higher total scores (i.e., summed item scores) on the RRES indicate higher levels of staff perceived rehabilitation engagement. The RRES was reported to have good inter-rater and test-retest reliability, as well as internal in its initial validation context (Meaden et al., 2012).

Analysis of RRES response patterns have supported the presence of three potential dimensions: ‘agreement with treatment and basic relationships’, ‘active participation and openness’, and ‘medication compliance’ (Meaden et al., 2012). The dimension ‘agreement with treatment and basic relationships’ included five items: 1a Relationship with the whole staff team; 1b. Relationship with the named nurse; 1c. Relationship with non-nursing; 5a. Will discuss and agree to rehabilitation interventions; and 5b. Will go along with rehabilitation if prompted. The dimension ‘active participation and openness’ includes ten items: 2a. Communication/openness regarding - personal feelings; 2b. Communication/openness regarding - problems/difficulties; 2c. Communication/openness regarding – challenging/risk behaviours; 3. Goal setting; 4. Perceived usefulness of rehabilitation goals; 5c. Active involvement with rehabilitation; 5d. Internal vs. external incentives re – involvement in rehabilitation; 5e. Completion of rehabilitation goals; and 6a. Appointment keeping without support. The dimension ‘medication compliance’ included a single item (7. Compliance with medication).

### Predictor Considerations

A few studies have examined predictors of poor engagement (including attrition) in mental health rehabilitation services and programs over the past two decades (see Table 1). Mixed findings have emerged in the literature with identified predictors including demographic variables (younger age (Arnautovska et al., 2021; Kurtz et al., 2011; Phalen et al., 2020), higher education level (Harding et al., 2008; Liu et al., 2023), relationship status (mixed findings: ever married (Liu et al., 2023)/never married (Harding et al., 2008)), illness-related variables (older onset (Liu et al., 2023), schizophrenia-spectrum disorder (Harding et al., 2008), higher alcohol use (Arnautovska et al., 2021), more positive psychotic symptoms (Harding et al., 2008), higher levels of health service utilization (Kurtz et al., 2011; Phalen et al., 2020), and program factors (Harding et al., 2008; Karan et al., 2022; Liu et al., 2023).

**Table 1:** Summary of studies considering predictors of poor engagement (including non-attendance and drop-out) in mental health rehabilitation

Study		Rehabilitation context	Service user information	Predictors of Poor Engagement ^a^
Arnautovska et al. (2021) Country: Australia Timeframe: 2014–2017		Community-based residential rehabilitation, recovery-oriented, TRR type	Engagement: *n* = 85/139 (61.15%, planned discharge) Diagnosis: SPMI (F20-29 *n* = 106/139, 76%) Age: 31.6 years ± 8.91 (at admission) Sex: Male *n* = 102/139 (73.4%)	Age: Younger Substance use: Higher alcohol Disability: Higher Recovery phase: Growth Significant trauma history: Yes ^b^
Harding et al. (2008) Country: USA Timeframe: 2008* * Publication year		Psychosocial rehabilitation program (supported employment and stepwise vocational program groups). Drop out is defined as exit within 6/12 of study commencement.	Engagement: *n* = 143/191 (73.71%, non-drop-out) Diagnosis: Schizophrenia spectrum disorders *n* = 116/194 (59.79%) Age: 38.7 years Sex: Male *n* = 122/194 (62.89%)	Education: Lower Relationship status: Never married Diagnosis: Schizophrenia-spectrum diagnosis Symptoms: More positive Sx Program: Stepwise model (vs. supported employment) NOT : substance use
Karan et al. (2022) Country: Australia Timeframe: 2021		Community-based residential rehabilitation, recovery-oriented, TRR type	Engagement: Agreement with proposed interventions (62.8%), and active participation (54.7%, *N* = 172) Diagnosis: F20-29 *n* = 83.1% (*n* = 143/172) Age: 34.3 years ± 11.07 Sex: Male *n* = 116/172 (64.7%)	Lower levels of agreement with proposed interventions and active involvement were noted in units not operating the integrated staffing model (where the majority of staff had a lived experience of mental illness).
Kurtz et al. (2011) Country: USA Timeframe: 2001–2008		Comprehensive rehabilitation treatment day program (3 days weekly). Focus on comprehensive cognitive training. Dropout was defined as < 10 h of computer training) or refusal of follow-up.	Engagement: *n* = 80/127 (62.99%, program completion) Diagnosis: Schizophrenia/schizoaffective disorder (100%) Age: 32.1 (SD 10.8) Sex: Male *n* = 98/127 (77.16%)	Age: Younger Cognition: Lower verbal fluency Interaction: Verbal fluency and illness duration. NOT: No relationship between clinical variables and dropout.
Liu et al. (2023) Country: Taiwan Timeframe: 2011–2018		Community-based, non-clinical, recovery-oriented support	Engagement: 139/162 (69.75%) Diagnosis: Severe mental illness (schizophrenia/schizoaffective *n* = 105/162) Age: Adult	Relationship status: Ever married Education: Lower level Illness onset: Older Referral: Outpatient/other (vs. day-centre) Vocational Hx: Work experience prior to onset
Phalen et al. (2020) Country: USA Timeframe: 2020* * Publication year		VA Medical Centres, 2x urban and one rural centre. The 12-session group-based intervention focused on health and wellness, intending to promote medical illness self-management behaviours among people with serious mental illness	Engagement: 0/12 sessions (17.7%), median = 6 (Mean 5.9 SD 4.4) Diagnosis: Schizophrenia/schizoaffective/Psychosis NOS *n* = 79/242 (32.64%) Age: = 57.75 ± 7.75 Gender: Male *n* = 210/242 (87%)	Age: Younger Service use: more ED visits in the six months prior Complexity: more medical conditions

^a^ Including attrition, drop-out, non-attendance

^b^ Self-initiated discharge is higher for people with a documented history of trauma

The 2023 CCU Benchmarking dataset included various demographic, illness-related, and health service use variables; including all known demographic, diagnostic, and illness-related predictors (except for illness onset). Data on current mental health symptoms were available from the 12-item Health of the Nation Outcome Scales (HoNOS) (Slade et al., 1999), and data on disability was available from the 16-item Life Skill Profile (LSP-16) (Trauer et al., 1995). The HoNOS and LSP-16 measures have considerable data supporting their reliability and validity (Burgess et al., 2017); both are mandatory outcome measures for public mental health services in Australia where their routine use is supported by national assessment and training processes (Burgess et al., 2015).

Comprehensive data on program factors, including staffing profiles and unit occupancy, staff recovery knowledge (as assessed by the Recovery Knowledge Inventory (RKI) (Bedregal et al., 2006), and treatment factors (e.g., involuntary treatment, chlorpromazine equivalence, long-acting injectable medication) were available. The full RKI instrument contains 20 items with a 5-point Likert scale, following reverse scoring of several items, higher scores equate to higher endorsement of recovery knowledge and attitudes. A 2023 systematic review of measures of recovery orientation for mental health staff identified that the RKI items covered the concepts of the CHIME (Connectedness, Hope and optimism, Identity, Meaning and purpose, Empowerment) Framework, had acceptable reliability *(a* = 0.72), but also issues with reliability and validity of 20-item version supporting the need for revision (Leamy et al., 2023). Given these challenges, we used the reduced 16-item RKI factor solution derived in an Australian mental health service context (Happell et al., 2015) in the analyses.

### Analysis

Analyses were completed in SPSSv30. Data was transformed and run through initial checks for linearity and unusual cases (see Supplementary Material for additional details of the modelling process). In presenting descriptive data at the participant level, the cohort was split based on the RRES total average score into those with an average score in the range of Never-to-Sometimes (score 1 ≤ 4.0) and those with an average score above ≥ 4 (Usually-to-Always). Differences between these groups were explored using inferential statistics (Chi-square for categorical variables and t-tests for continuous variables). The correlation matrix between potential predictors was analyzed to identify problematic variables (e.g., threats to the assumption of multi-collinearity and extreme outliers) and to rationalise variables for inclusion in the modelling. Following initial screening, and if relevant transformation and elimination of potential predictors, variables were entered simultaneously in the initial modelling round. Independent variables were included in the final models based on a threshold of *p* < .200 (Mickey & Greenland, 1989). The number of potential predictors included in the final model did not exceed the heuristic threshold of 10 cases per potential predictor (Field, 2018). The process for determining the adequacy of the modelling solution followed the procedure outlined by Field (Field, 2018). Interactions relating to time-related covariates (i.e., length of stay) were explored where relevant.

## Results

### Participant-level Data

208 participants met the inclusion criteria (see Table 2). The average age of participants was 36.78 years ($\stackrel{\sim}{x}$ = 35 years, SD = 11.48), and most were male (71.6%), born in Australia (83.7%), receiving a disability pension (59.6%), and had never been married (86.1%). Almost all participants were diagnosed with a schizophrenia spectrum disorder (ICD10 F20-29 diagnostic codes, 88.5%, F20.x = 66.3%), and more than a third had a concurrent non-tobacco substance use issue (33.7%). Most participants had been referred from a setting other than an acute inpatient mental health unit (61.1%). At the census date, the average length of stay was approximately 1-year ($\stackrel{-}{x}$ = 0.979, $\stackrel{\sim}{x}$ = 0.785, SD = 0.68 years), and most residents were subject to involuntary/community treatment orders (68.3%). All except for one participant were prescribed antipsychotic medication (chlorpromazine dose equivalents: $\stackrel{-}{x}$ = 487.96, $\stackrel{\sim}{x}$ = 425, SD = 325.32 mg), most were receiving antipsychotic polypharmacy (52.9%), and 45.7% were on a long-acting injectable formulation.

**Table 2:** Description of rehabilitation residents at the census date, with split based on residential rehabilitation (RRES) average total score: demographics, treatment, diagnosis, symptoms/impairment, engagement

Demographics		Total (*N* = 208)	Average RRES score	
			Never-to-Sometimes (< 4, 114/208 (54.8%))	Usually-to-Always (≥ 4, 94/208 (45.2%))
Age (years, (SD))		36.78 (11.48)	36.56 (11.80)	37.05 (11.14)
Male sex		71.6%	70.2%	73.4%
First Nations status		17.3%	23.7% ^*,a^	9.6%
Born outside Australia		16.3%	16.7%	16.0%
Never married		86.1%	84.2%	88.3%
Education ≤ 12 years		45.7%	42.1%	50.0%
Disability pension		59.6%	58.8%	60.6%
**Diagnosis**				
Schizophrenia-spectrum disorder ^b^		88.5%	88.6%	88.3%
Any non-tobacco substance use issue		33.7%	35.1%	31.9%
Multiple non-tobacco substance use issues		19.7%	20.2%	19.1%
Alcohol use disorder		10.6%	11.4%	9.6%
Nicotine use disorder/issue		61.1%	61.4%	60.6%
**Symptoms/impairment**				
Mental health symptoms (SD) ^c^		1.38 (0.79)	1.39 (0.81)	1.37 (0.77)
Positive psychotic symptoms (SD) ^c^		1.46 (1.18)	1.53 (1.24)	1.37 (1.11)
Cognitive impairment (SD) ^c^		1.22 (1.03)	1.29 (1.02)	1.13 (1.04)
Physical illness or disability (SD) ^c^		0.93 (1.08)	0.96 (1.17)	1.13 (1.04)
Agitation, aggression or disruptive behaviour (SD) ^c^		0.45 (0.77)	0.54 (0.86)	0.34 (0.63)
Non-accidental self-injury (SD) ^c^		0.10 (0.50)	0.06 (0.33)	0.15 (0.64)
Disability (LSP-16 average, (SD)) ^d^		0.89 (0.43) ^*,d^	1.02 (0.44)	0.73 (0.36)
**Treatment**				
Length of stay (years, (SD))		0.979 (0.677) ^*,e^	1.092 (0.728)	0.843 (0.584)
Involuntary treatment		68.3%	73.7%	61.7%
Referred from acute inpatient unit		38.9%	39.5%	38.3%
Chlorpromazine dose equivalence (mg, (SD))		487.96 (325.32)	473.66 (309.12)	505.31 (344.82)
Long-acting injectable antipsychotic		45.7%	41.2%	51.1%
Antipsychotic polypharmacy		52.9%	52.6%	53.2%
Clozapine prescribed		41.8%	43.0%	40.4%
Psychosocial interventions offered (of x/6 ^f^, (SD),)		4.24 (1.31), 4	4.33 (1.25), 4	4.13 (1.38), 4
Proportion offered accepted		82.2%	82.2%	82.1%
**Residential Rehabilitation Engagement Scale** ^g^				
Total average item score (SD)		3.81 (0.68) ^*,h^	3.30 (0.46)	4.42 (0.31)
Active participation average (SD)		3.60 (0.76) ^*,i^	3.06 (0.51)	4.26 (0.38)
Agreement & basic relationships average (SD)		4.08 (0.71) ^*,j^	3.59 (0.55)	4.66 (0.34)

^a^ First Nation status (i.e., identifying as Aboriginal and/or Torres Strait Islander): X^2^_(1)_ = 7.166, *p* = .007, Cramer’s V = 0.186

^b^ ICD10 F20-29 disorder as the primary psychiatric diagnosis

^c^ Derived from HoNOS (scores are scaled from 0 = ‘no problem’ to 4 = ‘severe or very severe problem’): Mental health symptoms = average of Items 6–8, Positive psychotic symptoms = Item 6; Cognitive impairment = Item 4, Physical impairment = Item 5, Agitation, aggression, or disruptive behaviour = Item 1, and Non-accidental self-injury = Item 2. Missing data was present: Items 6–8 (*n* = 2/208), Item 6 (*n *= 1/208), Item 4 (*n* = 1/208), and Item 1 (*n* = 1/208)

^d^ Average score across the 16 LSP-16 items, scaled (0 = ‘no difficulty’ to 3 = ‘extreme difficulty’: t_(1,202)_ = 5.082, *p* < .001, Cohen’s d = 0.88; note missing data (*n *= 4/208)

^e^ Length of stay: t_(1,206)_ = 5.082, *p* < .008, Cohen’s d = 0.66

^f^ Six psychosocial interventions were considered: Cognitive Behaviour Therapy, Cognitive Remediation, Family education and support, Psychoeducation, and Living skills development. Residents who were on the waitlist, the intervention was in progress, or it had been completed were counted as having been accepted. Except for ‘living skills interventions’ all other psychosocial interventions are classified in the ‘Royal Australian and New Zealand College of Psychiatrists clinical practice guidelines for the management of schizophrenia and related disorders’ (Galletly et al., 2016) as having ‘Level 1 evidence’ for schizophrenia

^g^ RRES (scaled scores with higher scores indicating higher levels of engagement (response range 1-to-5) average score: Cluster 1 = Active participation (Items: 2a-d, 3, 4, 5c-e, 6a); Cluster 2 = Agreement with treatment and basic relationships (Items: 1a-c, 5a-b); Cluster 3 = Compliance (single Item 7) is not presented

^h^ RRES Total Average: t_(1,206)_ = -20.145, *p* ≤ .001, Cohen’s d = 0.398

^i^ RRES ‘Active participation’: t_(1,206)_ = -18.812, *p *≤ .001, Cohen’s d = 0.459

^j^ RRES ‘Agreement with treatment and basic relationships’: t_(1,206)_ = −16.420, *p *≤ .001, Cohen’s d = 0.467

The average LSP-16 item score for participants was 0.886 (SD = 0.431); approximating a total score of 14.2 that exceeded the threshold for ‘high complexity’ (≥ 12) for the ‘functional gain’ phase and approached the threshold for ‘high complexity’ (≥ 15) for the ‘intensive extended’ phase as defined for community-based consumers in the Australian Mental Health Care Classification v1.0 (The Independent Health and Aged Care Pricing Authority, 2023). Regarding symptoms and impairments, the average total HoNOS score within the sample was 11.18 (SD = 5.76, $\stackrel{\sim}{x}$ = 10, range 0–35) which is within the ‘moderate severity’ level for people with severe mental illness (total score 10–12) proposed by Parabiaghi et al. (2005). The proportion of participants with recent clinically significant problems (assessed by HoNOS item score ≥2) relating to positive psychotic symptoms, cognitive impairment, and physical health issues, ‘overactive or aggressive or disruptive or agitated behaviours’, and non-accidental self-injury were 52.7%, 41.5%, and 28.4%, 9.7% and 2.4% respectively.

The average RRES item score across the cohort was 3.82 (SD = 0.68; item score ‘3’ = sometimes and ‘4’ = usually). Higher average scores on RRES items for the factor ‘Agreement and basic relationships’ (x̄ = 4.08 (SD: 0.71)) than for the factor ‘Active participation’ (x̄ = 3.60 (SD = 0.76)) were noted. Compared with participants with an average RRES score in the range of Usually-to-Always, those with an average score in the range of Never-to-Sometimes were more likely to identify as Aboriginal and/or Torres Strait Islander (First Nations status: 23.7 v 9.6%, X^2^_(1)_ = 7.166, *p* = .007, Cramer’s V = .186), have higher levels of disability (LSP-16 average: t_(1,202)_ = 5.082, *p* < .001, Cohen’s d = .88) and to have had a longer length of stay (t_(1,206)_ = 5.082, *p* < .008, Cohen’s d = .66).

### Unit-level Data

Occupancy levels at census data across the fourteen CCUs ranged from 70 to 97% ($\stackrel{\sim}{x}$ = 89%). The full-time equivalent unit staff-to-resident ratio averaged 1.02 (range: 0.83 − 1.40, $\stackrel{\sim}{x}$ = 0.99). Most units had PSWs available (n = 10/14; the proportion of staffing profile ranging from 0 to 69% ($\stackrel{\sim}{x}$ = 3%). The proportion of PSWs within the staffing profile at the integrated staffing model sites ranged from 28 to 69% ($\stackrel{\sim}{x}$ = 60%); for the non-integrated staffing sites, this ranged from 0 to 8% ($\stackrel{\sim}{x}$ = 1%). Regarding the RKI data, 225 staff responses were available, and the response rate per full-time equivalent staffing roles at each CCU ranged from 41 to 100% ($\stackrel{\sim}{x}$ = 77%). The average RKI score across the 16 items considered was 3.28 (SD = 0.50; scoring range 1-to-5), with the average scores for items considered under each factor being 3.21 (SD = 0.58) for ‘Roles and relationships’, 4.07 (SD = 0.53) for ‘Roles of self-definition and peers’, and 2.66 (SD = 0.76) for ‘Recovery as a non-linear process’. No differences were identified based on the unit staffing model (clinical/integrated/partnership) on the average total RKI-16 and subordinate factor scores.

### Multivariate Modelling – predictors of Residential Rehabilitation Engagement (staff rated)

The Supplementary Materials provide extensive details of the modelling decision-making process. The variables considered in the final multiple linear regression model were Age, First Nations status, HoNOS Item 5 (physical illness or disability), LSP-16 average score, Length of treatment, Integrated staffing model, and RKI average score. No diagnostic (including substance use) and medication-related variables were included.

The overall regression model was statistically significant (R^2^ = 0.290, adjusted R^2^ = 0.265, F_(7, 196)_ = 11.496, *p* < .001, see Table 3). Statistically significant predictors of higher levels of residential rehabilitation engagement (staff-rated) were: LSP-16 average score ($B$ = −0.413 (95% CI for B: − 0.872 to − 0.451), *p* < .001), length of treatment ($B$ = −0.165 (95% CI for B: − 0.296 to − 0.046), *p* = .008, Integrated staffing model ($B$ = 0.156 (95% CI for B: 0.064 to 0.501), *p* = .012), average RKI ($B$ = 0.138 (95% CI for B: 0.038 to 1.159), *p* = .037), and HoNOS Item 5 (physical illness or disability, $B$ = 0.129 (95% CI for B: 0.002 to 0.163), *p *= .045). Age ($B$ = 0.056, *p *= .374) and First Nations status ($B$ = −0.087, *p *= .158) did not significantly predict residential rehabilitation engagement.

**Table 3:** Multiple linear regression model of predictors of staff-rated mental health residential rehabilitation engagement (RRES-16 average score)

Predictor considerations (for dependent variable RRES-16 average score)^a,b^			B (SE)	$B$	95% CI for $\text{B}$			*p*^b^	
Type	Category	Variable							
Unit level	Staffing	Integrated staffing model	0.283 (0.111)	0.156	0.064	-	0.501	0.012	^*^
		Recovery Knowledge Inventory	0.599 (0.284)	0.138	0.038	-	1.159	0.037	^*^
Consumer level	Demographic	Age (years)	0.003 (0.004)	0.056	-0.004	-	0.011	0.374	
		First Nations status (yes)	-0.161 (0.113)	-0.087	-0.384	-	0.063	0.158	
	Symptoms and impairment	HoNOS Item 5 (Physical illness or disability)	0.083 (0.041)	0.129	0.002	-	0.163	0.045	^*^
		Average LSP-16 score (Disability)	-0.661 (0.107)	-0.413	-0.872	-	-0.451	< 0.001	^****^
	Service history	Length of treatment	-0.171 (0.063)	-0.165	-0.296	-	-0.046	0.008	^**^

^a^ Additional model details: simultaneous entry, R^2^ = 0.290, adjusted R^2^ = 0.265; F_(7, 196)_ = 11.496, *p* < 0.001; max VIF = 1.23 ($\overline{x }$ = 1.11); minimum tolerance statistic = 0.81 ($\overline{x }$ = 0.91)

^b^ ^*^ = *p* < 0.05, ^**^ = *p* < 0.05, ^****^ = *p* < 0.001

## Discussion

This study explored factors associated with staff-assessed resident engagement in residential mental health rehabilitation services using cross-sectional data from Queensland, Australia. Key strengths of this study included the comprehensive sampling approach and the availability of a broad range of predictor considerations, including both resident- and service-related variables. Less than half of the residents (45.2%) had an average RRES score consistent with them being ‘usually’ or ‘always’ engaged with the support available at the service. Resident and service level factors predicted higher levels of resident engagement. At the resident level, higher levels of rehabilitation engagement were associated with lower levels of psychosocial disability (LSP-16), higher levels of ‘physical illness or disability’ (HoNOS Item 5), and shorter length of stay at the rehabilitation unit. At the service level, higher levels of rehabilitation engagement were associated with the integrated staffing model (compared to the clinician and partnership approaches) and higher staff recovery knowledge and attitudes (RKI). Demographic, diagnostic, symptomatic, medication-related, and involuntary treatment (at the unit level) did not emerge as significant predictors of rehabilitation engagement.

The levels of engagement observed in this study were similar to those reported in the original study that applied the RRES to inpatient and community residential mental health rehabilitation services in the UK (Meaden et al., 2014). Additionally, like that study, we noted higher levels of engagement for the factor ‘Agreement and basic relationships’ than ‘Active participation’. Regarding the significant predictors of rehabilitation engagement in the final model, a single study conducted in the Queensland CCU context (Arnautovska et al., 2021) also reported an association between disability (as assessed by the independence-competence sub-scale of the Social Functioning Scale (Birchwood et al., 1990) and unplanned discharge. However, that study found that higher levels of disability were associated with a lower likelihood of unplanned discharge (which we had considered as a proxy for rehabilitation engagement) (Arnautovska et al., 2021). A single previous study also identified higher levels of medical comorbidity being associated with mental health rehabilitation engagement (Phalen et al., 2020). Length of stay was also identified as a predictor of rehabilitation engagement, with staff tending to rate levels of engagement lower for participants with a longer length of stay. In contrast, modeling of post-discharge outcomes for Queensland CCU residents has indicated that favorable outcomes are more likely for individuals with longer episodes of care. The service level predictor of receiving care under the integrated staffing model was consistent with data from an earlier benchmarking round (Karan et al., 2022). Additionally, the finding that higher recovery knowledge and attitudes were associated with improved engagement is consistent with a small body of literature supporting this variable being associated higher levels of resident satisfaction in rehabilitation contexts (Barrett et al., 2010; Parker et al., 2021).

Unlike previous studies that tended to focus on service-user factors, demographic (i.e., age (Arnautovska et al., 2021; Kurtz et al., 2011; Phalen et al., 2020), education level, and relationship status (Harding et al., 2008; Liu et al., 2023), diagnostic (e.g., schizophrenia spectrum disorders (Harding et al., 2008), alcohol use (Arnautovska et al., 2021), and symptom-related variables (i.e., positive psychotic symptoms (Harding et al., 2008), and cognitive impairment (Kurtz et al., 2011) did not emerge as significant predictors of rehabilitation engagement. The non-replication of several known potential predictors in the current study was not surprising given the small number of relevant studies and the heterogeneity of the rehabilitation contexts and predictor considerations. Additionally, the primary diagnoses within our sample were relatively homogenous, with 88.5% of people being diagnosed with a schizophrenia spectrum disorder.

The absence of alcohol and other substance use disorders predicting rehabilitation engagement was surprising given the emphasis in qualitative studies completed in the Queensland CCU context on the detrimental impact of substance use on readiness for rehabilitation engagement (staff perspective) (Parker et al., 2017) and sense of a positive community (resident perspective) (Parker et al., 2021). However, the finding aligns with substance use not emerging as a significant predictor of psychosocial outcomes following CCU rehabilitation support (Parker et al., 2020, 2023). This data suggests the relevance of optimism regarding rehabilitation engagement and outcomes for residents with concurrent substance use issues.

Residents with higher levels of psychosocial disability were less likely to be viewed by staff as being engaged with the rehabilitation service. While this is not an unexpected finding, it is an area that warrants further exploration, given that addressing the psychosocial disability associated with schizophrenia and related psychotic disorders is a primary goal of the CCU model.

Efforts to enhance service user experience and engagement with mental health services have included emphasis on principles of recovery-oriented care (Chatwiriyaphong et al., 2024; Gee et al., 2017; McKenna et al., 2016) and the increased availability of lived experience workers (e.g., the integrated staffing model) (Parker et al., 2016, 2023). The modelling exercise supported the potential value of these initiatives. Staffing models and staff recovery orientation are modifiable. In our sample, the integrated staffing model sites did not display higher recovery knowledge and attitudes than the other staffing models. This suggests that the higher staff ratings of resident rehabilitation engagement under the integrated staffing model were not simply a function of these staff having more optimistic attitudes towards residents. Previous qualitative studies have emphasised residents’ positive reflections on the availability of peer support workers under the integrated staffing model in the CCU context (Parker et al., 2021, 2025).

There are multiple potential explanations for the finding that residents with shorter rehabilitation durations had higher engagement levels. Residents who are well engaged may be likely to move on quickly to alterative accommodation in the community. Alternatively, residents with longer durations of care may remain at the CCU due to limitations in the availability of alternative accommodation and may have already completed the rehabilitation programs relevant to their needs.

### Limitations

The modelling is based on the secondary analysis of cross-sectional benchmarking data from a single jurisdiction and should be considered exploratory. It is not possible to draw causal inferences from the analysis. Limitations were present in the variables available for consideration. It is possible that a different modelling solution may have arisen with more rigorous data collection procedures, a larger sample size, and longitudinal data collection.

The lack of consistency in the literature meant that limiting the variable considerations to a small set of known predictors at the outset was inappropriate. Potential predictors of rehabilitation engagement in the literature that were not available in the data set included residents’ stage of personal recovery, trauma history, and duration since the onset of mental illness. Additionally, other variables of potential relevance such as the negative symptoms of psychosis and therapeutic alliance were not available. Furthermore, data on involuntary treatment were limited to the unit level rather than at the level of each resident.

The inclusion of data at both the individual resident and unit level in the modelling means that people experiencing care under each unit are likely more similar than a random sample across the population. As such, the assumption that each person’s data is independent of the others was not strictly met. Replication of our findings in similar contexts is needed to increase confidence, and in alternative rehabilitation settings to support generalizability.

The RRES is a staff-rated measure of residential rehabilitation engagement and does not provide an objective measure of a resident’s engagement. This outcome focus reflects the perspective of a single stakeholder and misses the perspectives of consumers. Furthermore, information about the rater(s) who completed the RRES for each CCU site is unavailable. Additionally, including a focus on ‘medication compliance’ is inconsistent with contemporary clinical language, which focuses on collaboration between residents and prescribers (e.g. ‘adherence or ‘concordance’ (Rae, 2021).

## Conclusion

This study provides useful insights to support ongoing efforts to better engage consumers residing at mental health rehabilitation units. Notably, the modelling exercise identified modifiable factors associated with increased rehabilitation engagement available to mental health services. The association between higher levels of staff recovery knowledge and attitudes supports the continuing effort to enhance the recovery orientation of mental health staff through training and cultural change. Additionally, the association between receiving care under the integrated staffing model, where peer support worker availability is prioritized, and enhanced engagement provides support for ongoing exploration and evaluation of similar service model changes to enhance the experience and outcomes of care. The findings are expected to enhance our understanding of the factors associated with rehabilitation engagement, guiding future initiatives to enhance resident participation in evidence-based psychosocial rehabilitation program.

## Supplementary Information

Below is the link to the electronic supplementary material.

Supplementary Material 1 (DOCX. 596 KB)

## Data Availability

Further release of data associated with this project would require approval from the relevant Human Research Ethics Committee and data custodian. The corresponding author is available to support such requests.
