# Supplementary material for: Predictors of Engagement in Community-based Residential Mental Health Rehabilitation: Modelling of a cross-sectional Statewide Benchmarking Dataset from Queensland, Australia
Source: Community Ment Health J. 2025 Sep 10;62(1):166–76. doi: 10.1007/s10597-025-01512-6 (PMC12789195; doi:10.1007/s10597-025-01512-6)
Supplement: Supplementary file 1 — Supplementary Material 1 (DOCX. 596 KB) [file 10597_2025_1512_MOESM1_ESM.pdf]

Predictors of engagement in community-based residential mental health rehabilitation: Modelling of a cross-sectional statewide benchmarking dataset from Queensland, Australia

SUPPLEMENTARY MATERIAL

21st July 2025

Contents

1: Additional information about the Queensland CCU context 3
1a. Service model description 3
1a-1. Historical overview (inception to 2019) 3
1a-2. Staffing models 3
1a-3. Contemporary outcomes 4
1b. Consumer perspectives 4
1b-1. Post-discharge experience 4
1b-2. Rehabilitation support experience 4
1b-3. Consumer expectations 5
1c. Staff experience 5
1d. Variation between staffing models 6
2: Variables available for consideration 8
2a: Factors and covariates for consideration, including known and novel predictors. 8
2b: Resident-level variable considerations (before rationalisation) 9
2c: Unit-level variable considerations (before rationalisation) 10
3. Rationalisation of variables for consideration 11
3a: Identifying optimal variables to consider construct domains based on correlation matrices ... 12
3b: Reduced variable set, assessing multicollinearity. 15
3c: Outlier detection based on the reduced variable set (2b). 16
3d: Rationalising covariate/factor considerations based on a p<.20 decision rule. 17
3e: Reduced variable set, assessment for multicollinearity and outliers. 18
3f: Review of the final proposed variable set, incl. assessment for multi-collinearity and outliers. 19
3g: Additional consideration of HoNOS Items 1 and 2 based on peer review feedback 21
References 22

1: Additional information about the Queensland CCU context

1a. Service model description

1a-1. Historical overview (inception to 2019)

Source: Parker, S., Hopkins, G., Siskind, D. *et al.* A systematic review of service models and evidence relating to the clinically operated community-based residential mental health rehabilitation for adults with severe and persisting mental illness in Australia. *BMC Psychiatry* 19, 55 (2019). https://doi.org/10.1186/s12888-019-2019-5 [1]

- Describes the change over time from a focus on residential support to the provision of transitional residential rehabilitation that is time-limited with the expectation of assisting consumers to move-on followed the focused provision of recovery-oriented rehabilitation support.
- Identifies the consistent focus on supporting people experiencing severe and persisting mental illness (predominantly schizophrenia) with complex care needs.

[Link to open access]

1a-2. Staffing models

Source: Parker, S., Dark, F., Vilic, G., McCann, K., O'Sullivan, R., Doyle, C. and Lendich, B. (2016), "Integrated staffing model for residential mental health rehabilitation", Mental Health and Social Inclusion, Vol. 20 No. 2, pp. 92-100. https://doi.org/10.1108/MHSI-12-2015-0043. [2]

- Description and rationale for the integrated staffing model approach where most staff at the clinically operated rehabilitation service have a lived experience of mental illness and recovery.

[Link to article Abstract, not open access]

Source: Parker S, Dark F, Newman E, Korman N, Meurk C, Siskind D, Harris M. Longitudinal comparative evaluation of the equivalence of an integrated peer-support and clinical staffing model for residential mental health rehabilitation: a mixed methods protocol incorporating multiple stakeholder perspectives. BMC psychiatry 2016; 16: 179. 2016/06/04. DOI: 10.1186/s12888-016-0882-x. [3]

- Additional information about the rationale for the integrated staffing model, including historical context.
- Provides an overview (now dated) of the challenges historically experienced in incorporating lived experience roles within routine clinical mental health care.

[Link to open access]

Source: Meurk, C., Parker, S., Newman, E. and Dark, F., 2019. Staff expectations of an Australian integrated model of residential rehabilitation for people with severe and persisting mental illness: a pragmatic grounded theory analysis. Frontiers in Psychiatry, 10, p.468. [4]

- Explores what lived experience and clinical staff expect from working together under the integrated staffing model.
- Optimism expressed about the service models potential but also uncertainty about how this will work in practice.

[Link to open access]

1a-3. Contemporary outcomes

Source: Parker, S., Arnautovska, U., Siskind, D., Dark, F., McKeon, G., Korman, N., and Harris, M. (2020). Community-care unit model of residential mental health rehabilitation services in Queensland, Australia: predicting outcomes of consumers 1-year post discharge. Epidemiology and Psychiatric Sciences 29 e109 1-11. https://doi.org/10.1017/s2045796020000207 [5]

- Queensland statewide administrative data for the period 2005-2014.
- Many consumers experienced reliable improvements in relevant outcomes following CCU support.
- Those with poorer mental health and social functioning (at admission) and longer periods of CCU care were more likely to make RCS improvements in mental health and social functioning.

[Link to Open Access]

1b. Consumer perspectives

1b-1. Post-discharge experience

Source: Parker, S, Chapman, M., Wyder, M. et al. Life is better but not without challenges: experiences following discharge from community-based residential mental health rehabilitation–a qualitative content analysis. Soc Psychiatry Psychiatr Epidemiol (2024). [Access: https://doi.org/10.1007/s00127-024-02716-z] [6]

- Considers integrated and clinical staffing model consumers. Absence of marked differences in reflections (which were positive) on the impact of CCU care on post-CCU life.

Link: Life is better but not without challenges: experiences following discharge from community-based residential mental health rehabilitation–a qualitative content analysis | Social Psychiatry and Psychiatric Epidemiology

1b-2. Rehabilitation support experience

Source: Parker, S., Wyder, M., Pommeranz, M., Newman, E., Meurk, C. and Dark, F. 2021. Consumer experiences of community-based residential mental health rehabilitation for severe and persistent mental illness: A pragmatic grounded theory analysis. Int J Mental Health Nurs, 30(3): 733-746. [7]

- Interviews exploring consumer experiences under the integrated and clinical staffing models.
- There were no major differences in themes emerging, but whose under the integrated model emphasized valuing the availability of lived experience workers.

[Link to Open Access]

1b-3. Consumer expectations

Source: Parker S, Meurk C, Newman E, Fletcher C, Swinson I, Dark F. Understanding consumers' initial expectations of community-based residential mental health rehabilitation in the context of past experiences of care: A mixed-methods pragmatic grounded theory analysis. International journal of mental health nursing 2018; 27: 1650-1660. 2018/04/18. DOI: 10.1111/inm.12461. [8]

- Exploration of consumer expectations on commencement (both integrated and clinical sites) of rehabilitation care in comparison to historical care. Lack of direct focus on staffing approach but clear emphasis from consumers on issues suggesting lack of past recovery-oriented care historically.
- Expectations aligned with principles articulated in policy frameworks for recovery-oriented practice.

[Link to open access]

Source: Parker S, Dark F, Newman E, et al. Consumers' understanding and expectations of a community-based recovery-oriented mental health rehabilitation unit: a pragmatic grounded theory analysis. Epidemiology and psychiatric sciences 2019; 28: 408-417. 2017/12/05. DOI: 10.1017/s2045796017000749. [9]

- Absence of meaningful differences in consumer expectations between those commencing at integrated and clinically staffed sites.
- Consumers understand the function of the rehabilitation service, but most commencing identify limited accommodation options rather than the availability of rehabilitation support as a reason for choosing to come.

[Link to article Abstract, not open access]

1c. Staff experience

Source: Parker, S., Dark, F., Newman, E. et al. Staff Experiences of Integrating Peer Support Workers and Clinical Staff in Community-Based Residential Mental Health

Rehabilitation: A Pragmatic Grounded Theory Analysis. Community Ment Health J (2023). [10]

- Explores the implementation experience of the integrated staffing model from the perspectives of staff.
- Note positive reflections from both clinical and lived experience staff, shared learning, enhancement of rehabilitation practices.

[Link to Abstract, not open access]

Source: Wyder, M., Roennfeldt, H., Parker, S., Vilic, G., McCann, K., Ehrlich, C., & Dark, F. L. (2020). Diary of a Mental Health Peer Worker: Findings From a Diary Study Into the Role of Peer Work in a Clinical Mental Health Setting. Frontiers in Psychiatry, 1282. [11]

- Exploration of what the work of a peer worker is in a residential rehabilitation setting from the perspectives of lived experience workers.
- Lived experience workers emphasized flexible, responsive and adaptive ways of working in support of residents.
- Viewed their work as supporting a different perspective and facilitating more personal ways of working for the rehabilitation team (i.e., clinical staff).

[Link to Open Access]

Source: Parker S, Dark F, Newman E, Korman N, Rasmussen Z, Meurk C. Reality of working in a community-based, recovery-oriented mental health rehabilitation unit: A pragmatic grounded theory analysis. Int J Ment Health Nurs. 2017 Aug;26(4):355-365. doi: 10.1111/inm.12251. Epub 2016 Sep 3. PMID: 27589881. [12]

- Clinical staff model staff experience from a single site.
- Four themes identified: ‘rehabilitation is different to treatment’; ‘the CCU is a positive transitional space’; ‘they (consumers) have to be ready to engage’; and ‘recovery is central to rehabilitation practice’.
- Identified threats to recovery-oriented practice included burnout and acceptance of consumers to the unit who were not ready to engage.

[Link to Open Access]

1d. Variation between staffing models

Source: Parker, S., Arnautovska, U., Korman, N. et al. Comparative Effectiveness of Integrated Peer Support and Clinical Staffing Models for Community-Based Residential Mental Health Rehabilitation: A Prospective Observational Study. Community Ment Health J 59, 459–470 (2023). https://doi.org/10.1007/s10597-022-01023-8 [13]

- This paper identified an absence of meaningful differences in clinical outcomes between the clinical and integrated staffing models, but where differences were present there was a suggestion that these favored the integrated approach.

[Link to Abstract, not open access]

Source: Lalley N, Jones D, Stedman T, Parker S. Does fundamentally altering the staffing of clinical rehabilitation services impact their function? Australasian Psychiatry. 2023;0(0). doi:10.1177/10398562231189427 [14]

- Cross sectional study identifying differences in who receives care based on the staffing configurations (i.e., reduced acuity and inpatient referrals under the integrated approach compared to the clinical).

[Access link]

Source: Karan N, Parker S, Jones D, Stedman T. 2022. Cross-Sectional Comparison of Treatment Provided Under the Clinical, Integrated, and Partnership Staffing Models for Community-Based Residential Mental Health Rehabilitation. Community Ment Health J., 58(5), 907-916. [Access: https://doi.org/10.1007/s10597-021-00898-3.] [15]

- Cross sectional study suggesting differences in treatment between the integrated and clinical models despite lack of difference in consumer characteristics. The nature of these differences suggested potential higher alignment under the integrated model with recovery-oriented care.

[Link to Abstract, not open access]

Source: Parker S, Siskind D, Hermens DF, et al. A Comprehensive Cohort Description and Statistical Grouping of Community-Based Residential Rehabilitation Service Users in Australia. Frontiers in psychiatry 2019; 10: 798. 2019/11/30. DOI: 10.3389/fpsyt.2019.00798. [16]

- Noted minimal site-based variability in the characteristics of consumers admitted to CCU operating a clinical and integrated staffing model (i.e., reduction in clinical staff availability not changing accepted consumer profile).

[Link to open access]

2: Variables available for consideration

2a: Factors and covariates for consideration, including known and novel predictors.

SERVICE USER LEVEL			Known	Source	Available [a]
**Demographics**	**Age (older)**		Yes	[17-19]	Yes
	**Education level (lower)**		Yes	[20, 21]	Yes
	**Relationship status (mixed findings)**		Yes	[20, 21]	Yes
	**First Nations status**		-	-	Yes
	**Overseas born / non-English speaking background**		-	-	Yes
**Illness**	**Diagnosis**	**Schizophrenia spectrum disorders**	Yes	[20]	Yes
		**Higher alcohol use**	Yes	[17]	Yes
		**Other substance use issues**	-	-	Yes
		**Older onset**	Yes	[21]	No
	**Symptoms**	**Positive psychotic symptoms (higher)**	Yes	[20]	Yes
		**Cognitive impairment (verbal fluency, higher)**	Yes	[18]	Yes
	**Impairment**	**Disability (higher)**	Yes	[17]	Yes
	**Medical comorbidity/complexity**		Yes	[19]	Yes
**Personal**	**Recovery stage (growth)**		Yes	[17]	No
	**Trauma history (present)**		Yes	[17]	No
**Service History**	**Referral source (non-outpatient)**		Yes	[21]	Yes
	**Length of stay**		-	-	Yes
	**Involuntary treatment (any)**		-	-	Yes
	**History of more Emergency Department visits**		Yes	[19]	No
**Medication**	**Chlorpromazine dose equivalence**		-	-	Yes
	**LAI prescribed**		-	-	Yes
	**Clozapine prescribed**		-	-	Yes
**UNIT LEVEL**			**Known**	**Source**	**Available**
**General**	**Distance from CBD**		-	-	Yes
	**Relative socioeconomic advantage**		-	-	Yes
	**Occupancy at census (%)**		-	-	Yes
**Staffing**	**Integrated staffing model (peer support worker availability)**		Yes	**[22]**	Yes
	**Staff-to-resident ratio**		-	-	Yes
	**RKI**		-	-	Yes
**Cohort**	**Proportion of involuntary residents (%)**		-	-	Yes
**Program**	**Stepwise program (cf. supported employment)**		Yes	[20]	N/A

[a] Including approximations and/or related data

2b: Resident-level variable considerations (before rationalisation)

**RESIDENT-LEVEL**			**Variable consideration(s)**	**Source(s)**
**Demographics**	**Age (older)**		Age at census	Administrative data extraction
	**Education level (lower)**		Education level (Scaled)	Administrative data extraction
	**Relationship status (mixed findings)**		Ever married (including de facto, separated, married, divorced, widowed) (Yes/No)	Administrative data extraction
	**First Nations status**		Aboriginal and/or Torres Strait Islander (Yes/No)	Administrative data extraction
	**Overseas born / non-English speaking background**		Born in Australia (Yes/No)	Administrative data extraction
**Illness**	**Diagnosis**	**Schizophrenia spectrum disorders**	ICD10 F20-29 disorder (Yes/No)<br>ICD10 F20.x disorder (Yes/No)<br>ICD10 F25.x disorder (Yes/No)	Administrative data extraction
		**Higher alcohol use**	ICD10 F10.x disorder (Yes/No)	Administrative data extraction
		**Other substance use issues**	Any non-nicotine related substance use issue (ICD10-16.x or F18-19.x : Yes/No)<br>Any nicotine-related substance use issue (ICD10 F17.x: Yes/No)<br>Multiple non-F17.x substance use issues (>1, excluding F17.x in F10-19.x: Yes/No)	Administrative data extraction and/or<br>Key worker benchmarking survey
	**Symptoms**	**Positive psychotic symptoms (higher)**	HoNOS Items 6-8 average (mental health symptoms, general)<br>HoNOS Item 6 (positive symptoms)	Administrative data extraction
		**Cognitive impairment (verbal fluency, higher)**	HoNOS Item 4 (cognitive impairments)	Administrative data extraction
	**Impairment**	**Disability (higher)**	LSP-16 average	Administrative data extraction
	**Medical comorbidity/complexity**		HoNOS Item 5 (physical impairments)	Administrative data extraction
**Service History**	**Referral source (non-outpatient)**		Acute mental health inpatient referral (Yes/No)	Key worker benchmarking survey
	**Length of stay**		CCU length of stay at census date	Administrative data extraction
	**Involuntary treatment (any)**		Any involuntary treatment at census date (Yes/No)	Administrative data extraction
	**History of more Emergency Department visits**		-	Administrative data extraction
**Medication**	**Chlorpromazine dose equivalence**		Chlorpromazine dose equivalence (mg)	Key worker benchmarking survey
	**LAI prescribed**		Long-acting injectable antipsychotic prescribed (Yes/No)	Key worker benchmarking survey
	**Antipsychotic polypharmacy**		>1 antipsychotic medications prescribed (Yes/No)	Key worker benchmarking survey
	**Clozapine prescribed**		Clozapine prescribed (Yes/No)<br>Clozapine dose (mg)	Key worker benchmarking survey

2c: Unit-level variable considerations (before rationalisation)

UNIT LEVEL		Variable consideration(s)	Source(s)
**General**	**Distance from CBD**	Distance from capital city CBD in km	Unit postcode
	**Relative socioeconomic advantage**	SEIFA index of socio-economic advantage	Unit postcode
	**CCU site**	The 13 individual sites included in the dataset	Key worker benchmarking survey
**Staffing**	**Integrated staffing model (peer support worker availability)**	Integrated staffing model (Yes/No)<br>Proportion of actual staffing in Peer Support Worker roles (%)	Team leader/NUM unit level benchmarking survey
	**Staff-to-resident ratio**	Staff to consumer ratio (at census date, %)	Team leader/NUM unit level benchmarking survey
	**Recovery Knowledge (using RKI** [23])	RKI20 average for unit staff<br>RKI16 average for unit staff<br>RKI16 Factor 1 (roles and responsibilities) – average for unit staff<br>RKI16 Factor 2 (roles of self-definition and peers) – average for unit staff<br>RKI16 Factor 3 (recovery as non-linear process) – average for unit staff	All staff benchmarking survey (anonymous)
**Cohort**	**Proportion of involuntary residents (%)**	Proportion of cohort subject to involuntary treatment at census (%)	Administrative data extraction

3. Rationalisation of variables for consideration

Process:

	**Step**	**Key observations/actions taken**
1	Visualisation by scatterplots to consider linearity and identify unusual cases ([variable] * RRES/PropInt/IntOffAcceptAll).	Removal of cases with a LOS <42 days (0.115 years) as these residents will likely be in the assessment rather than the interventional phase of care, limiting rehabilitation engagement opportunities.<br>Removal of cases with a LOS of >3 years, which is 50% higher than the designated length of stay as per the model of service. These, by definition, reflect atypical cases.<br>Decision made transform the variable outcome 'Proportion of interventions offered accepted' to dichotomous variable 'All interventions offered accepted (Yes/No)'. There was a disproportionate number of 100% values (53%) and n=6/205 0% values (3%).
2	Examination of differences between CCU sites on the primary and secondary outcome foci.	ANOVA identified Site 5 as an outlier with significantly higher RRES average scores than Sites 3 (p=.028) and 12 (p=0.25), and on post hoc comparisons, it approaches significance in comparison to Site 4 (p=.05). In Step 7 potential problematic cases were explored, none of these arose from Site 5.
3	Identify optimal variables for each target construct where multiple related variables were available for consideration and/or novel predictors are being considered.	See Supplementary Material 2a
4	Review of the correlation matrix of a reduced set of predictor considerations.	See Supplementary Material 2b
5	Exploration to identify outliers based on the reduced predictor consideration set (2b).	See Supplementary Material 2c
6	Rationalisation of variables for inclusion from reduced predictor consideration set (2c).	See Supplementary Material 2d.
7	Reduced variable set, including assessments for multicollinearity and outliers.	See Supplementary Material 2e.
8	Review of the final proposed variable set, including assessment for multi-collinearity and outliers.	See Supplementary Material 2f.<br>.

3a: Identifying optimal variables to consider construct domains based on correlation matrices

RESIDENT>DEMOGRAPHIC CONSIDERATIONS

Variable consideration	Known	RRES16 [a]	Concerns	Comment	Carry forward
Age at census	Yes	ns	Yes	Known predictor, no significant bivariate correlation	Y
Education level (scaled)	Yes	ns	Yes	Known predictor, no significant bivariate correlation	Y
Ever married	Yes	ns	Yes	Known predictor, no significant bivariate correlation	Y
Aboriginal and/or Torres Strait Islander	No	p=.033	No	Negative correlation with born outside of Australia	Y
Born outside of Australia	No	ns	No	-	N

RESIDENT>ILLNESS>DIAGNOSIS CONSIDERATIONS

Variable consideration	Known	RRES16 [a]	Concerns	Comment	Carry forward
ICD10 F20-29 disorder (Yes/No)	Yes	ns	Yes	ICD10 F20-29 is a known predictor but has no significant bivariate correlation with the outcome considered. The other two considerations reflect sub-categories, all are highly inter-correlated (expected). Therefore, only ICD10 F20-29 to carry forwards.	Y
ICD10 F20.x disorder (Yes/No)	(see above)	ns	Yes		N
ICD10 F25.x disorder (Yes/No)	(see above)	ns	Yes		N
ICD10 F10.x disorder (Yes/No)	Yes	ns	Yes	There is a significant inter-correlation between all substance use variables. Given that the known predictor (ICD10 F10.x disorder) was the only consideration with a significant bivariate correlation (only with IntOffAcceptAll), this was the only one in this set carried forward.	Y
ICD10-16.x or F18-19.x (Yes/No)	No	ns	Yes		N
ICD10 F17.x (Yes/No)	No	ns	Yes		N
>1, excluding F17.x in F10-19.x (Yes/No)	No	ns	Yes		N

[a] Bivariate correlation with p>.10 are reported as ‘ns’, note all Known predictors are carried forward

RESIDENT>ILLNESS>SYMPTOMS/IMPAIRMENT CONSIDERATIONS

Variable consideration	Known	RRES16 [a]	Concerns	Comment	Carry forward
HoNOS Items 6-8 (MH symptoms) average	No	p=.045	Yes	Positive psychotic symptoms is the known predictors, however the overall symptoms subscale for HoNOS was more highly correlated. Plan to carry both forward but only allow one in final model.	Y
HoNOS Item 6 (positive symptoms)	Yes	p=.088	Yes		Y
HoNOS Item 4 (cognitive impairments)	Yes	p=.024	No	Note that LSP-16 correlates with all other symptom/impairment considerations, but this is not unacceptably high. Similarly, intercorrelations are present between HoNOS items 4 and 5.	Y
HoNOS Item 5 (physical impairments)	Yes	ns	No		Y
LSP16 average	Yes	p<.001	No		Y

RESIDENT>SERVICE HISTORY & MEDICATIONS

Variable consideration	Known	RRES16 [a]	Concerns	Comment	Carry forward
Referral source (non-outpatient: Yes/No)	Y	ns	No	Note the correlation with both LAI and clozapine prescribed.	Y
Length of stay	N	p=.005	No	Note the correlations with any involuntary treatment (RRES).	Y
Involuntary treatment (any: Yes/No)	N	ns	Yes	Correlation with referral source and med variables (except cloz dose).	N
Chlorpromazine dose equivalent (mg)	N	ns	Yes	Note correlation with all other medication variables (expected).	N
LAI prescribed	N	ns	Yes	Correlated with antipsychotic polypharmacy.	N
Clozapine prescribed (Yes/No)	N	ns	Yes	Correlated with antipsychotic polypharmacy and CPZ eq.	N
Clozapine dose (mg)	N	ns	No	Correlated with CPZ eq (expected).	N

[a] Bivariate correlation with p>.10 are reported as 'ns', note all Known predictors are carried forward

UNIT>GENERAL & STAFFING

Variable consideration	Known	RRES16 [a]	Concerns	Comment	Carry forward
Distance from capital city CBD in km	No	p=.002	Yes	Correlated with RKI variables and proportion involuntary. [b]	Y
SEIFA index - socio-economic advantage	No	ns	No	Correlated with staffing, involuntary Tx, and RKI variables. [b]	N
CCU site	No	-	-	See Supplementary Material 2, Step 2.	Y
Integrated staffing model (Yes/No)	Yes	p=.065	Yes	Correlated with staff : consumer ratio, prop. Involuntary. [c]	Y
Proportion of PSW roles (%)	(see above)	ns	No	Expected correlation with Integrated Model.	N
Staff-to-consumer ratio	No	ns	No	Negative correlations with RKI variables noted.	N
RKI20 average for unit staff	No	p<.001	Yes	Note expected inter-correlations between RKI variables. Correlations between these and RRES were higher for RKI16 than the RKI20 (not RKI16 was an Australian validation) and higher for RKI16 F1. The decision was made to carry RKI16 and all RKI16 factor scores through to the next modelling stage but to use the RKI16 total average score of individual factor score(s) if included in the final modelling.	N
RKI16 average for unit staff	No	p<.001	Yes		Y
RKI16 Factor 1	No	p<.001	Yes		Y
RKI16 Factor 2	No	p<.001	Yes		Y
RKI16 Factor 3	No	p<.001	Yes		Y
Cohort – proportion involuntary (%)	No	p<.001	No	See above re correlations with integrated model and prop PSW.	Y

[a] Bivariate correlation with p>.10 are reported as 'ns'; note all Known predictors are carried forward

[b] Also, correlated with all other unit-level variables considered

[c] Also, correlated with other unit-level variables considered except RKI20 and 16 total and RKI16 F1

3b: Reduced variable set, assessing multicollinearity.

Group	Variable consideration	Known	Comments [a]	Carry forward
Outcome	RRES Total Average	N/A	-	Yes
Demographic	Age at census	Yes	-	Yes
	Education level (scaled)	Yes	-	Yes
	Ever married	Yes	-	Yes
	Aboriginal and/or Torres Strait Islander	No	-	Yes
Diagnosis	ICD10 F20-29 disorder (Yes/No)	Yes	-	Yes
	ICD10 F10.x disorder (Yes/No)	Yes	-	Yes
Impairment	HoNOS Items 6-8 (MH symptoms) average	No	As HoNOS item 6 is a component of HoNOS Items 6-8, both are not to be included in the final model for either outcome consideration. However, both will be evaluated further.	Yes
	HoNOS Item 6 (positive symptoms)	Yes		Yes
	HoNOS Item 4 (cognitive impairments)	Yes	-	Yes
	HoNOS Item 5 (physical impairments)	Yes	-	Yes
	LSP16 average	Yes	-	Yes
Treatment	Referral source (non-outpatient: Yes/No)	Y	-	Yes
	Length of stay	N	-	Yes
Unit	Distance from capital city CBD in km	N	-	Yes
	CCU site	N/A	The decision to examine the impact of the outlier site (Site 5) on a final model is already documented.	Yes
	Integrated staffing model (Yes/No)	Y	-	Yes
	RKI16 average for unit staff	N	Correlation with RKI F1 = .951 (p<.001), F2 = .617 (p<.001), and RKI F3 =.867 (p<.001).	Yes
	RKI16 Factor 1	N	See above.	Yes
	RKI16 Factor 2	N	See above.	Yes
	RKI16 Factor 3	N	See above	Yes
	Cohort – proportion involuntary (%)	N	-	Yes

[a] Correlation matrix examined to identify variables with r=/>.80

3c: Outlier detection based on the reduced variable set (2b).

The Mahalanobis distance was calculated for the 15 scaled variables (predictor considerations) included in the reduced variable set (2b) of relevance to the primary outcome focus RRES). No cases were identified with a probability ≤.001.

3d: Rationalising covariate/factor considerations based on a p<.20 decision rule.

Initial considerations:

Group	Variable consideration	RRES		Carry forward
		A	B	
**Demographic**	Age at census	p<.2	p<.2	Yes
	Education level (scaled)	ns	ns	No
	Ever married	p<.2	ns	No
	Aboriginal and/or Torres Strait Islander	ns	p<.2	Yes
**Diagnosis**	ICD10 F20-29 disorder (Yes/No)	ns	ns	No
	ICD10 F10.x disorder (Yes/No)	ns	ns	No
**Impairment**	HoNOS Items 6-8 (MH symptoms) average	ns	ns	No
	HoNOS Item 6 (positive symptoms)	ns	ns	No
	HoNOS Item 4 (cognitive impairments)	ns	ns	No
	HoNOS Item 5 (physical impairments)	p<.1	p<.1	Yes
	LSP16 average	p<.001	p<.001	Yes
**Treatment**	Referral source (non-outpatient: Yes/No)	ns	ns	No
	Length of stay	p<.01	p<.01	Yes
**Unit**	Distance from capital city CBD in km	ns	ns	No
	Integrated staffing model (Yes/No)	p<.2	p<.1	Yes
	RKI16 average for unit staff	N/A	p<.2	Yes
	RKI16 Factor 1	ns	N/A	No
	RKI16 Factor 2	ns	N/A	No
	RKI16 Factor 3	ns	N/A	No
	Cohort – proportion involuntary (%)	ns	ns	No

^ Set 'A' Considered the RKI16 Total average score, and set 'B' considered each factor individually; the decision was made to carry forward Set B given the signal for the RKI average for unit staff (and lack thereof for the individual Factors for this measure).

3e: Reduced variable set, assessment for multicollinearity and outliers.

Group	Variable consideration [a]	Type	Model A (n=204)	Model B (n=202)[b]	Model C (n=198)[c]	Comments
**Demographic**	Age at census	Continuous	p=.374	p=.442	p=.559	Removal of outliers under Model B and C does not alter the direction or meaningfully change the significance value for any of the predictor considerations. While HoNOS item 5 (physical impairments) shifted from p<.05 to p<.1 between Model A and B, the actual change was minimal, and Model C was consistent with Model A.
	Aboriginal and/or Torres Strait Islander	Dichotomous	p<.2	p<.2	p<.2	
**Impairment**	HoNOS Item 5 (physical impairments)	Scaled	p<.05	p<.1[d]	p<.05	
	LSP16 average	Continuous	p<.001	p<.001	p<.001	
**Treatment**	Length of stay	Continuous	p<.01	p<.01	p<.001	
**Unit**	Integrated staffing model (Yes/No)	Dichotomous	p<.05	p<.05	p<.01	
	RKI16 average for unit staff	Continuous	p<.05	p<.05	p<.05	
**Outcome**	RRES Total Average	Continuous	[redacted]			

[a] No significant interactions were identified between the predictors using the PROCESS macro in SPSS
[b] Excluding cases 202 and 203 (see Casewise diagnostics)
[c] Excluding cases 5-8 and 202-203 (see Casewise diagnostics)
[d] For 'Model A' B=.126, p=.045, for 'Model B' B=.127, p=.052, for 'Model C' B=.132, p=036

Assessing multicollinearity:

- All VIF scores were well below 10 (Max = 1.23, average = 1.107)
- All tolerance statistics were well above 0.2 (Min = .813, average = .907)
- Collinearity diagnostics showed each predictor loading onto a different dimension.
- These assessments raised no significant concerns.

Casewise diagnostics:

- Only 7 of the 204 included cases (3.4%) had a standardised residual <-2 or >2. Only 3 cases had standardised residuals <-2.5 or >2.5 (-2.652, -2.522, +2,756, n=3/204, 1.4%).
- No cases had a Cooks's distance >1 (i.e., undue influence on the model, Max=0.054).
- Exploration of the Mahalanobis distance identified 2 cases with a p<.01 (based on Chi square distribution, with df=6, threshold of 16.81): 18.56 & 20.42 (n2/204, >1%).
- DFBeta statistics were all within the range of -1-to-1.
- Covariance ratio was examined to detect cases with scores < .88 or > 1.12 (threshold based on 1(+/-)[3(7+1)/204]). Nine cases exceeded this threshold (n=9/204, 4.4%).
- Potentially influential cases were:

Case no	Site	Mahalanobis distance	Cook's distance	Covariance ratio	Consideration
005	4	9.45	.05430	.787	Across the potential influential cases, Cook's distance values suggest no major issues of concern. However, the decision was made to test the robustness of the model's findings by excluding: (1) Cases 203 & 204; and (2) All nine cases identified as potentially problematic on the coavariance ratios.<br><br>The impact of excluding potential problematic cases (Model B and Model C) are presented above. Given the absence of a meaningful impact of these exclusions on the model, all cases were retained.<br><br>NONE of the potential problematic cases arose from Site 5.
006	12	2.40	.01520	.787	
007	13	4.40	.02231	.816	
008	13	3.13	.01453	.843	
200	13	12.7	.00018	1.118	
201	2	14.23	.00002	1.126	
202	11	16.80	.00228	1.133	
203	4	18.56	.00482	1.136	
204	7	20.41	.00125	1.161	

3f: Review of the final proposed variable set, incl. assessment for multi-collinearity and outliers.

Group	Variable consideration [a]	Type	Model A (n=204)	Model B (n=202)[b]	Model C (n=198)[c]	Comments
**Demographic**	Age at census	Continuous	p=.374	p=.442	p=.559	Removal of outliers under Model B and C does not alter the direction or meaningfully change the significance value for any of the predictor considerations. While HoNOS item 5 (physical impairments) shifted from p<.05 to p<.1 between Model A and B, the actual change was minimal, and Model C was consistent with Model A.
	Aboriginal and/or Torres Strait Islander	Dichotomous	p<.2	p<.2	p<.2	
**Impairment**	HoNOS Item 5 (physical impairments)	Scaled	p<.05	p<.1[d]	p<.05	
	LSP16 average	Continuous	p<.001	p<.001	p<.001	
**Treatment**	Length of stay	Continuous	p<.01	p<.01	p<.001	
**Unit**	Integrated staffing model (Yes/No)	Dichotomous	p<.05	p<.05	p<.01	
	RKI16 average for unit staff	Continuous	p<.05	p<.05	p<.05	
**Outcome**	RRES Total Average	Continuous	N/A			

[a] No significant interactions were identified between the predictors using the PROCESS macro in SPSS
[b] Excluding cases 202 and 203 (see Casewise diagnostics)
[c] Excluding cases 5-8 and 202-203 (see Casewise diagnostics)
[d] For 'Model A' B=.126, p=.045, for 'Model B' B=.127, p=.052, for 'Model C' B=.132, p=036

Assessing multicollinearity:

- All VIF scores were well below 10 (Max = 1.23, average = 1.107)
- All tolerance statistics were well above 0.2 (Min = .813, average = .907)
- Collinearity diagnostics showed each predictor loading onto a different dimension.
- These assessments raised no significant concerns.

Casewise diagnostics:

- Only 7 of the 204 included cases (3.4%) had a standardised residual <-2 or >2. Only 3 cases had standardised residuals <-2.5 or >2.5 (-2.652, -2.522, +2,756, n=3/204, 1.4%).
- No cases had a Cooks's distance >1 (i.e., undue influence on the model, Max=0.054).
- Exploration of the Mahalanobis distance identified 2 cases with a p<.01 (based on Chi square distribution, with df=6, threshold of 16.81): 18.56 & 20.42 (n2/204, >1%).
- DFBeta statistics were all within the range of -1-to-1.
- Covariance ratio was examined to detect cases with scores < .88 or > 1.12 (threshold based on 1(+/-)[3(7+1)/204]). Nine cases exceeded this threshold (1.61, 1.14, 1.13, 1.13, 1.12, .84, .82, .79, 79; n=9/204, 4.4%).
- Potentially influential cases were:

Case no	Site	Mahalanobis distance	Cook's distance	Covariance ratio	Consideration
005	4	9.45	.05430	.787	Across the potential influential cases, Cook's distance values suggest no major issues of concern. However, the decision was made to test the robustness of the model's findings to excluding: (1) Cases 203 & 204; and (2) Cases 5-to-8 and 203-204.<br><br>The impact of excluding potential problematic cases (Model B and Model C) are presented above. Given the absence of a meaningful impact of these exclusions on the model, all cases were retained.<br><br>NONE of the potential problematic cases arose from Site 5.
006	12	2.40	.01520	.787	
007	13	4.40	.02231	.816	
008	13	3.13	.01453	.843	
200	13	12.7	.00018	1.118	
201	2	14.23	.00002	1.126	
202	11	16.80	.00228	1.133	
203	4	18.56	.00482	1.136	
204	7	20.41	.00125	1.161	

Final model (for dependent variable RRES-16 average score) [a,b]			B (SE)	*B*	95% CI for B			P [b]	
Type	Category	Variable							
Unit level	Staffing	Integrated staffing model	.283 (.111)	.156	.064	-	.501	.012	*
		Recovery Knowledge Inventory	.599 (.284)	.138	.038	-	1.159	.037	*
Consumer level	Demographic	Age (years)	.003 (.004)	.056	-.004	-	.011	.374	
		First Nations status (yes)	.161 (.113)	.087	-.063	-	.384	.158	
	Symptoms and impairment	HoNOS Item 5 (Physical illness or disability)	.083 (.041)	.129	.002	-	.163	.045	*
		Average LSP-16 score (Disability)	-.661 (.107)	-.413	-.872	-	-.451	<.001	****
	Service history	Length of treatment	-.171 (.063)	-.165	-.296	-	-.046	.008	**

[a] Additional model details: simultaneous entry, R^2=.290, adjusted R^2=.265; $F_{(7, 196)}$=11.496, p<.001; max VIF = 1.23 ($\bar{x}$=1.11); minimum tolerance statistic = .81 ($\bar{x}$=.91)

[b] * = p<.05, ** = p<.05, **** = p<.001

3g: Additional consideration of HoNOS Items 1 and 2 based on peer review feedback

A query was raised about the relevance of reporting the full set of behavioural problems available in the HoNOS scale (i.e., additional inclusion of Items 1 and 2).

These variables were not included in the original planned predictor considerations based on the scoping of the literature.

Further consideration of these variables identified:

[1] **Low frequency of clinically significant issues for these items within the sample**

HoNOS Item 1 with score ≥2 = 9.7% (n=18/207)

HoNOS Item 2 with score ≥2 (2.4%, n=5/208).

[2] **A significant bivariate correlation between HoNOS Item 1 and RRES (average)**: r=-.209, p=.003

[3] **Absence of significant bivariate correlation between HoNOS Item 2 and RRES (average)**: r=.040, p=.564

Based on very low frequency of clinically significant issues relating to HoNOS Item 2 and the absence of significant bivariate correlation with RRES (average), no further consideration was made to the impact on the final modelling solution.

Acknowledging the low frequency of clinically significant issues relating to HoNOS Item 1, but the presence of a significant bivariate correlation with RRES (average), the impact of including this variable on the final model was considered.

**Exploration of impact of inclusion of HoNOS 1 on Final model (DV= RRES-16 average score)** [a,b]			**B (SE)**	***B***	**P** [b]
**Type**	**Category**	**Variable**			
Unit level	Staffing	Integrated staffing model	.276 (.112)	.153	.014
		Recovery Knowledge Inventory	.571 (.288)	.132	.049
Consumer level	Demographic	Age (years)	.003 (.004)	-.049	.436
		First Nations status (yes)	-.165 (.114)	-.089	.149
	Symptoms and impairment	HoNOS Item 1	-.016 (.060)	-.018	.786
		HoNOS Item 5 (Physical illness or disability)	.083 (.041)	.129	.045
		Average LSP-16 score (Disability)	-.664 (.116)	-.410	<.001
	Service history	Length of treatment	-.172 (.064)	-.166	.007

The inclusion of this variable **<u>did not change</u>** the modelling solution and HoNOS 1 did not emerge as a significant predictor.

Hence **<u>the original final model was retained</u>**.

References

1. Parker, S., et al., *A systematic review of service models and evidence relating to the clinically operated community-based residential mental health rehabilitation for adults with severe and persisting mental illness in Australia.* BMC Psychiatry, 2019. **19**(1): p. 55.
2. Parker, S., et al., *Integrated staffing model for residential mental health rehabilitation.* Mental Health and Social Inclusion, 2016. **20**(2): p. 92-100.
3. Parker, S., et al., *Longitudinal comparative evaluation of the equivalence of an integrated peer-support and clinical staffing model for residential mental health rehabilitation: a mixed methods protocol incorporating multiple stakeholder perspectives.* BMC Psychiatry, 2016. **16**(1): p. 179.
4. Meurk, C., et al., *Staff Expectations of an Australian Integrated Model of Residential Rehabilitation for People With Severe and Persisting Mental Illness: A Pragmatic Grounded Theory Analysis.* Frontiers in Psychiatry, 2019. **Volume 10 - 2019**.
5. Parker, S., et al., *Community-care unit model of residential mental health rehabilitation services in Queensland, Australia: predicting outcomes of consumers 1-year post discharge.* Epidemiology and Psychiatric Sciences, 2020. **29**: p. e109.
6. Parker, S., et al., *Life is better but not without challenges: experiences following discharge from community-based residential mental health rehabilitation–a qualitative content analysis.* Social Psychiatry and Psychiatric Epidemiology, 2025. **60**(1): p. 95-111.
7. Parker, S., et al., *Consumer experiences of community-based residential mental health rehabilitation for severe and persistent mental illness: A pragmatic grounded theory analysis.* International Journal of Mental Health Nursing, 2021. **30**(3): p. 733-746.
8. Parker, S., et al., *Understanding consumers' initial expectations of community-based residential mental health rehabilitation in the context of past experiences of care: A mixed-methods pragmatic grounded theory analysis.* International Journal of Mental Health Nursing, 2018. **27**(6): p. 1650-1660.
9. Parker, S., et al., *Consumers' understanding and expectations of a community-based recovery-oriented mental health rehabilitation unit: a pragmatic grounded theory analysis.* Epidemiology and Psychiatric Sciences, 2019. **28**(4): p. 408-417.
10. Parker, S., et al., *Staff Experiences of Integrating Peer Support Workers and Clinical Staff in Community-Based Residential Mental Health Rehabilitation: A Pragmatic Grounded Theory Analysis.* Community Mental Health Journal, 2023. **59**(4): p. 703-718.
11. Wyder, M., et al., *Diary of a Mental Health Peer Worker: Findings From a Diary Study Into the Role of Peer Work in a Clinical Mental Health Setting.* Frontiers in Psychiatry, 2020. **Volume 11 - 2020**.
12. Parker, S., et al., *Reality of working in a community-based, recovery-oriented mental health rehabilitation unit: A pragmatic grounded theory analysis.* International Journal of Mental Health Nursing, 2017. **26**(4): p. 355-365.
13. Parker, S., et al., *Comparative Effectiveness of Integrated Peer Support and Clinical Staffing Models for Community-Based Residential Mental Health Rehabilitation: A Prospective Observational Study.* Community Mental Health Journal, 2023. **59**(3): p. 459-470.
14. Lalley, N., et al., *Does fundamentally altering the staffing of clinical rehabilitation services impact their function?* Australas Psychiatry, 2023. **31**(5): p. 706-707.
15. Karan, N., et al., *Cross-sectional comparison of treatment provided under the clinical, integrated, and partnership staffing models for community-based residential mental health rehabilitation.* Community Mental Health Journal, 2022. **58**(5): p. 907-916.
16. Parker, S., et al., *A Comprehensive Cohort Description and Statistical Grouping of Community-Based Residential Rehabilitation Service Users in Australia.* Front Psychiatry, 2019. **10**: p. 798.

17. Arnautovska, U., et al., *Predictors of unplanned discharge from community-based residential mental health rehabilitation for people affected by severe and persistent mental illness.* J Ment Health, 2021. **30**(4): p. 500-508.
18. Kurtz, M.M., J. Rose, and B.E. Wexler, *Predictors of participation in community outpatient psychosocial rehabilitation in schizophrenia.* Community Ment Health J, 2011. **47**(6): p. 622-7.
19. Phalen, P.L., et al., *Predictors of attendance in health and wellness treatment groups for people with serious mental illness.* Psychiatr Rehabil J, 2020. **43**(2): p. 149-155.
20. Harding, B., et al., *Factors associated with early attrition from psychosocial rehabilitation programs.* Community Ment Health J, 2008. **44**(4): p. 283-8.
21. Liu, Y.-C., et al., *Predictors of Participation and Effectiveness in Community-based Psychiatric Rehabilitation Program in People with Severe Mental Illness: psychiatric rehabilitation utilization.* International Journal of Child Development and Mental Health, 2023. **11**(1): p. 1-19.
22. Karan, N., et al., *Cross-Sectional Comparison of Treatment Provided Under the Clinical, Integrated, and Partnership Staffing Models for Community-Based Residential Mental Health Rehabilitation.* Community Ment Health J, 2022. **58**(5): p. 907-916.
23. Bedregal, L.E., M. O'Connell, and L. Davidson, *The Recovery Knowledge Inventory: assessment of mental health staff knowledge and attitudes about recovery.* Psychiatric rehabilitation journal, 2006. **30**(2): p. 96.
